# Evaluating construction parameters of HB and CD methods for super large section tunnel: a case study

**DOI:** 10.1038/s41598-023-42458-7

**Published:** 2023-09-22

**Authors:** Ting Zhu, Yuanming Liu

**Affiliations:** Faculty of Civil Engineering, Guizhou University, Guiyang, 550025 China

**Keywords:** Civil engineering, Energy infrastructure, Mechanical engineering

## Abstract

The double sidewall guide pit and centre cross diagram (CRD) methods are often used for the construction of large-section tunnels through water-rich fault fracture zones due to their long construction time and high construction cost. To shorten the construction period and save costs, the top heading and benching method (HB) and centre diaphragm (CD) can be chosen for construction. The construction parameters of the step and CD methods are optimized to ensure the surrounding rock stability and tunnel safety**.** By relying on the Tongzi Tunnel, we simulate the excavation of different step heights in the construction of the top heading and benching method (HB) and CD methods through numerical simulation, the laws of tunnel vault settlement, and changes in the surrounding rock stress, initial support axial force, bending moment and safety factor. The study shows that as the height of the upper step increases, the settlement of the vault and the convergence of the periphery increase, the initial support safety factor decreases, and the plastic zone of the surrounding rock increases at 30 m from the target face. The step height for the top heading and benching method (HB) of construction is optimized as follows. The ratios of the upper, middle and lower step heights are 0.45H, 0.35H and 0.2H, respectively. The CD method construction step height is optimized to the left (right) upper and left (right) lower step height ratios of 0.5H and 0.5H, respectively.

## Introduction

With the continuous development of China's economy, the construction of highways in Guizhou has been expanding; however, when building highways, tunnels are inevitably constructed because they can effectively reduce the mileage, slope and curvature, thus improving the operational efficiency. During the construction of fractured surrounding rock (Class V surrounding rock), serious deformation damage often occurs due to complex stresses and the uneven release of surrounding rock stress^1,2^. Therefore, the reasonable use of the excavation method can effectively reduce the construction disturbance and control the stress and strain of the surrounding tunnel rock well, which can guarantee the stability of the project^3–5^. For large span tunnels in coal strata with soft rock and prominent gas and large deformation tunnels in soft rock with high ground stress, the problem of efficient safety is abrupt^6–11^. The three steps method, the CD method, the CRD method and the double sidewall guide trench method are common techniques for tunnelling in soft and fractured rock. Among these techniques, the three three steps and the CD method have short construction periods, low costs and convenient construction, but they are prone to large deformation and collapse accidents. Therefore, the optimization of the geometric parameters of the three-stage method of construction has been a popular issue studied by experts and scholars^12–16^.

In response to the above problems, global scholars have performed much research. Xu and Wang^17^, relying on the Guanghuai (Tong)-Dali railroad Nanhua 1 tunnel project, studied the deformation, damage and the stress disturbance characteristics of the typical red-layered soft surrounding tunnel rock in central Yunnan through model tests; additionally, they derived the deformation, damage and stress change characteristics of the surrounding rock caused by tunnel excavation in the shallow buried section. Wei^18^, relying on the Beijing Tunnel Project as the research background, studied the problem of sudden mud collapse of the tunnel through indoor model tests; the factors influencing the instability of the surrounding rock and the damage law and influence range of the tunnel have been derived. Liu et al.^19^ studied the change law of the internal stress of the existing surrounding tunnel rock, the internal displacement of the surrounding rock and the internal force of the support structure caused by the excavation of the new tunnel through model tests. In this paper, systematic research and analysis on the optimization of step geometry parameters is carried out to propose the optimal geometry parameters for the construction of tunnels through the soft crushed zone top heading and benching method (HB). Duan and Yang^20^ used MIDAS GTS List of numerical-analysis software to analyse the influences of three bench excavation footage on the stability of large section shallow buried tunnel, and they concluded that the tunnel excavation is stable when the excavation footage is not more than 1.5 m. Ma^21^ used ABAQUS finite element software to simulate and analyse the tunnel scheme of the three-line station under complex geological conditions and to optimize the construction parameters of the double sidewall guide pit method, which effectively controlled the development of surrounding tunnel rock deformation and ensured construction safety. Zhu^22^ used numerical analysis software to simulate large section tunnels using circuitous excavation means; optimization research work was carried out on the construction step distance and support parameters of the double sidewall guide pit method. Yi^23^ studied the mechanical characteristics and applicability of the bench method construction for large span tunnels in soft rock. It is noted that the engineering geological conditions should be carefully analysed and evaluated in the construction of the top heading and benching method (HB). Cao and Zhu^24^ conducted a numerical simulation of crack propagation using 3DEC, and the propagation patterns of surface cracks under various burial depth conditions were obtained. Luo et al.^25^ noted that the three-step and seven-step excavation methods should increase the size of the core soil while shortening the length of the middle and lower steps. Yang^26^ proposed the optimization construction measures of setting up the core soil of the upper step, replacing the middle partition of the lower step with temporary steel pipes, and expanding the area of the lower step. Li et al.^27^ conducted a model test study on the failure of the lining in various parts of a super large cross-section deep buried tunnel and proposed construction measures to strengthen the strengths of the tunnel sidewalls and arch lining.

Although certain achievements have been made globally in the study of the deformation and damage characteristics of tunnels crossing complex sections of soft surrounding rocks, further research is needed to optimize the construction methods. In this paper, based on the Tongzi tunnel project, the optimization scheme of the construction parameters of the top heading and benching method (HB) is derived through numerical analysis, which provides a reference for the design and construction of similar projects in the future.

## Project overview

**Figure 1:**
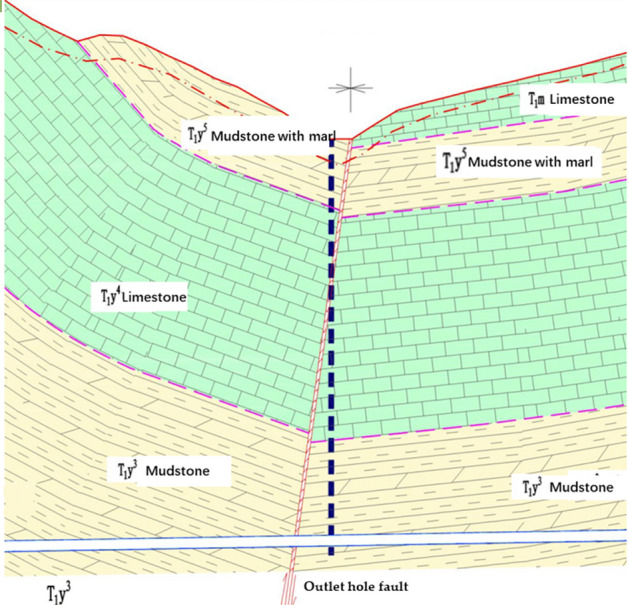
Longitudinal section.

**Figure 2:**
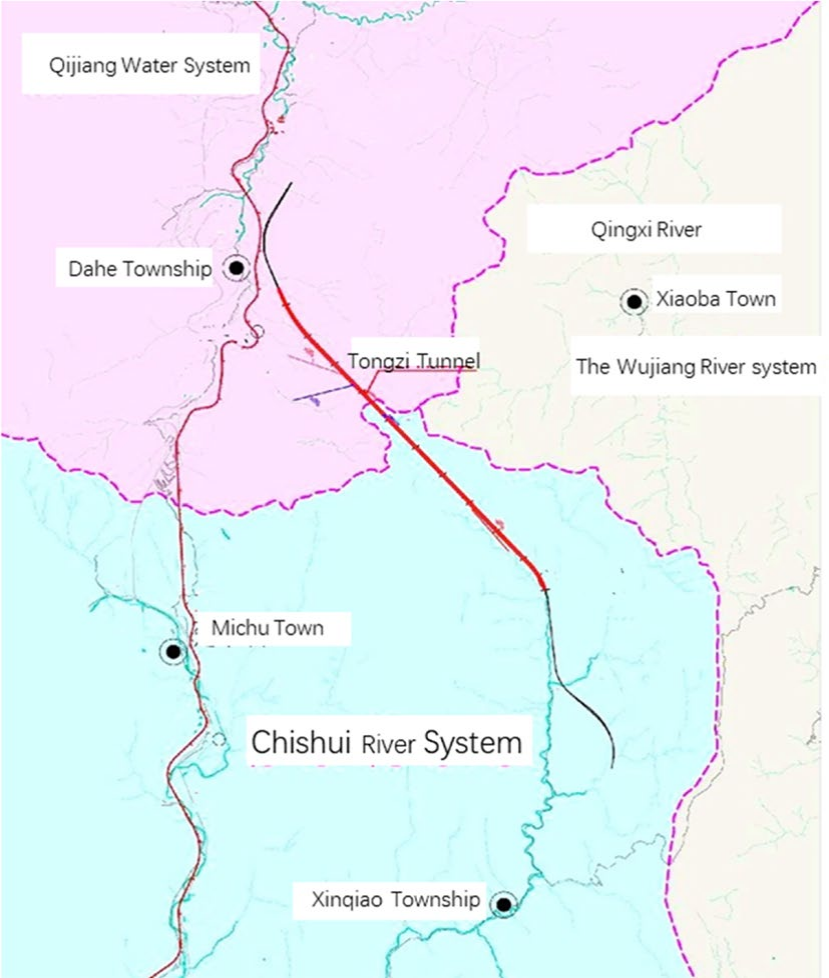
Water system sketch.

The Lanhai Expressway K0 + 000 ~ K119 + 285.1 line length is 109.069 km. The starting point is in the village of Xiaping at the junction of Chongqing and Chongqing, and the end point connects to the under construction expansion of Zungui. Tongzi Tunnel is in Tongzi County, Guizhou, in the heavy Zun section of Lanhai Expressway. The project area is in the northern plateau of Qian, crossing the Great Lou Mountains with large topographic changes. The lowest point is in the Songkan River valley with an elevation of 405.0 m. The highest point is at the ridge of Tongzi Tunnel with an elevation of 1646.4 m and a relative height difference of 1241.4 m. The landform type is mainly dissolution and erosion of the Zhongshan landform. The tunnel crosses the fold structure of the East Mountain backslope, the high bridge oblique, and the Maoba oblique; the fracture is mainly open shoulder Fort fault, Ling Hu Jia Pass fault, water hole fault and night cat cave fault. The tunnel is divided into two townships in Tongzi County, with the central watershed of the tunnel as the boundary. The Chongqing end is under the jurisdiction of the Dahe township, and the Zunyi end is under the jurisdiction of the Mahair township. The Tongzhi Tunnel is a separated long tunnel. The starting and ending pile numbers on the left are ZK34 + 508 ~ ZK45 + 005, the total length is 10,497 m, and the maximum depth is approximately 639.61 m. The starting and ending pile numbers on the right are YK34 + 530 ~ YK45 + 015, the total length is 10,485 m, and the maximum depth is 639.07 m. The line spacings between the left and right sides of the tunnel are approximately 20.9 m for the inlet section and 21.9 m for the outlet section. The longitudinal section of the Tongzi tunnel outlet hole fault is shown in Fig. 1, and the water system in the Tongzi tunnel site area is shown in Fig. 2.

**Figure 3:**
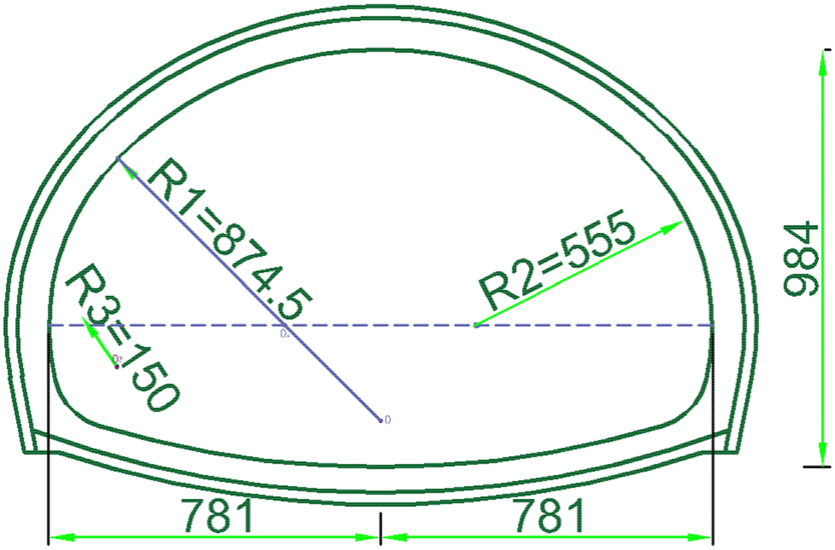
Tunnel cross section (Unit: cm).

In this paper, we select the water-rich fault fracture zone out of the water hole fault section for the study; the starting and ending mileage pile numbers range from ZK42 + 250 to ZK42 + 330, the total length is 80 m, and the top plate burial depth is 346–387 m. The cave surrounding rock is medium weathering mudstone, which is strongly affected by the fault; the rock body is broken, and the tunnel excavation may produce rain-like outflow water. Groundwater is abundant, and the surrounding rock is not self-stabilizing; thus, it is easy to produce slumping and falling blocks when there is no support. The system is supported according to the V level surrounding rock. The cross-sectional view of the tunnel is shown in Fig. 3 below.

The surrounding rocks exposed at the palm face of the Tongzi Tunnel are mainly medium-weathering mudstone, which is extremely weak and fragmented, and rich in groundwater, which is prone to collapse, water gushing and support structure cracking damage during excavation. Therefore, to ensure the safety levels of construction personnel and construction equipment, it is necessary to optimize the construction parameters of the soft and fractured surrounding rock section to reduce the large deformation of the surrounding rock to better guide engineering design and construction.

## Calculation model and parameters

The MIDAS/GTS NX numerical analysis software was used to establish a numerical analysis model for the Tongzi tunnel section from ZK42 + 250 to ZK42 + 330, which has a total length of 80 m and an average tunnel depth of 366 m. The model parameters are taken according to the geological exploration report of Tongzi and Tunnel Design Specification for Highway Tunnels (JTG 3370.1-2018). The parameters of the surrounding rock are shown in Table 1, the parameters of the anchor rods are shown in Table 2 and the parameters of the initial concrete spraying support are shown in Table 3.

**Figure 4:**
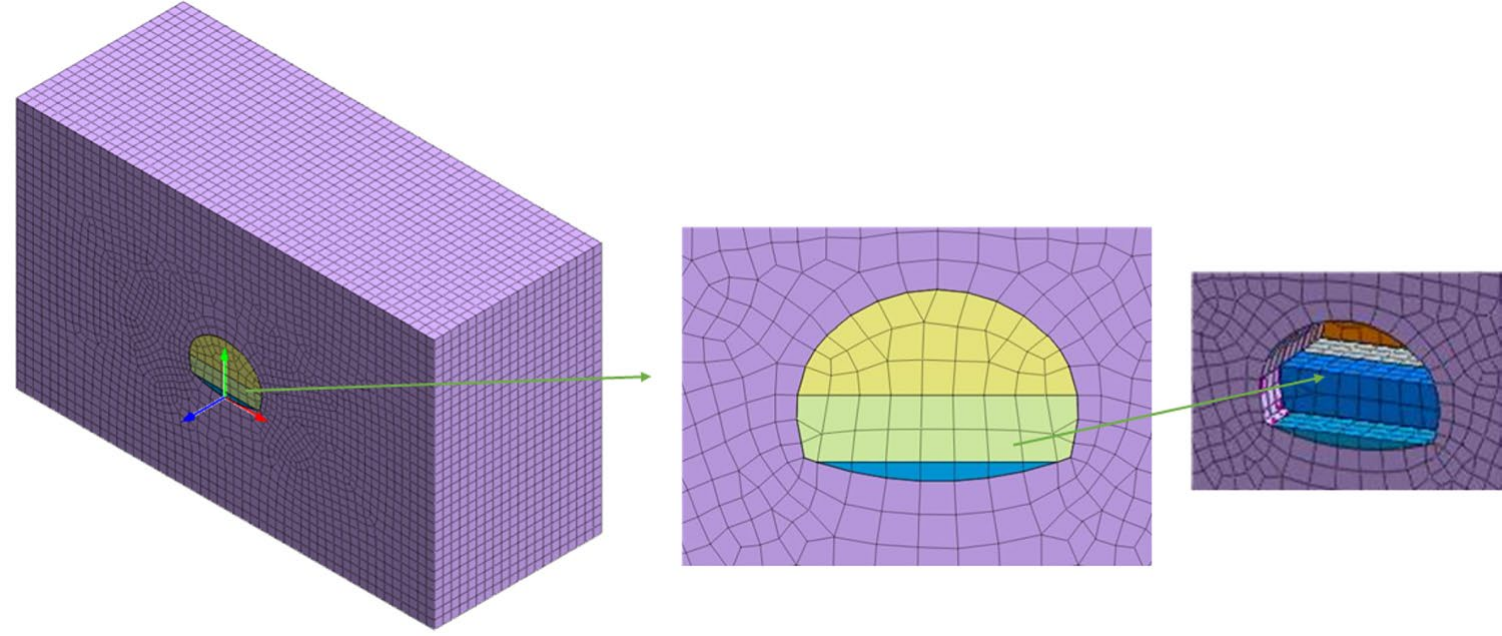
Construction model of the top heading and benching method (HB).

**Figure 5:**
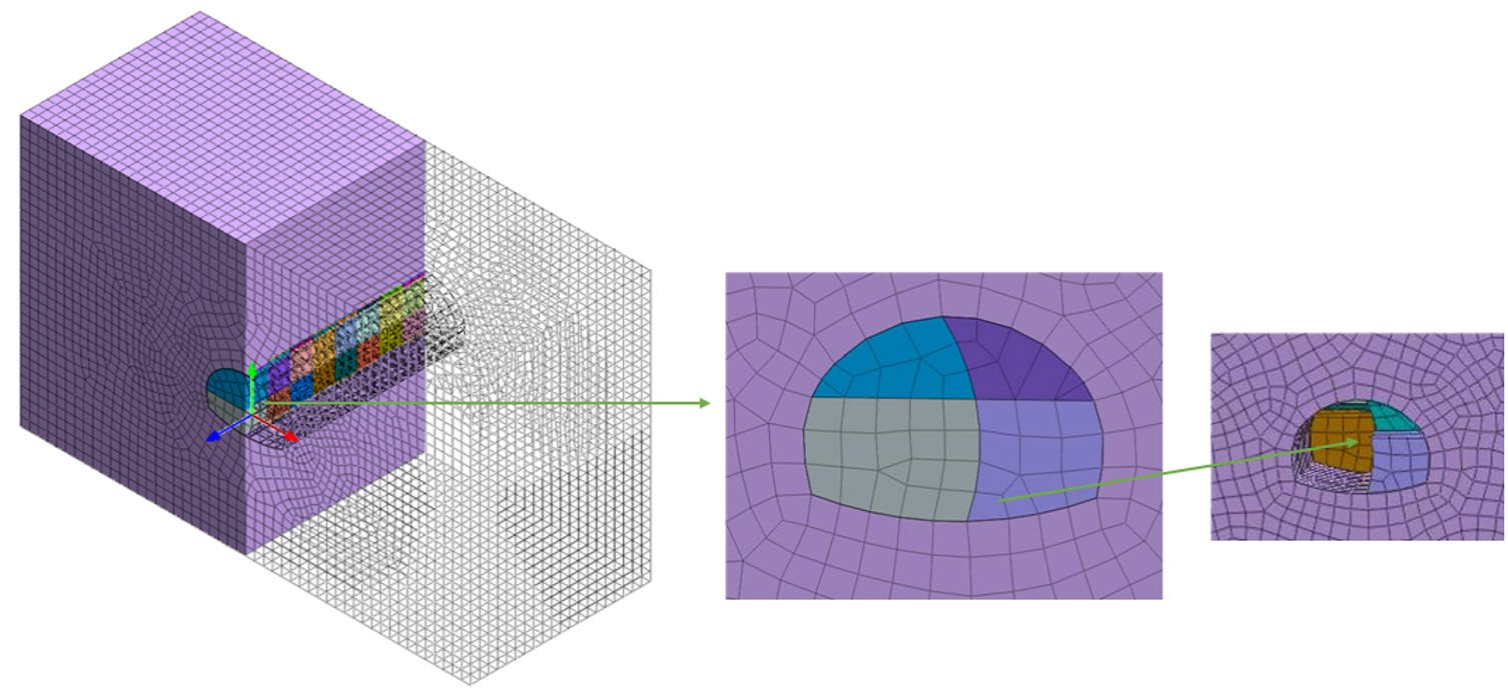
CD method construction model.

1. The calculations consider that the rock mass follows the ideal elastic‒plastic model and that the yield condition is the Mohr Coulomb criterion.
2. The finite element model is 100 m × 80 m × 60 m in length, width and height, respectively, and the model contains 37,546 nodes and 40,606 units. The initial support (shotcrete) adopts the slab unit, and the rock body adopts the solid unit. The construction models of the top heading and benching method (HB) and CD method are shown in Fig. 4 and Fig. 5 below.
3. The boundary conditions are as follows: a normal constraint perpendicular to the face is added at the bottom. The outer interface is the perimeter of the surrounding rock plus the normal restraint of the vertical surrounding rock face. The upper boundary is the load-free boundary.

**Table 1:** List of surrounding rock parameters.

Surrounding rock	$\gamma$(KN/m^3^)	Modulus of elasticity, E (GPa)	Poisson’s ratio, $\mu$	Cohesion, c (MPa)	Friction angle, $\varphi$ (°)
V	17	1.1	0.42	0.12	23.5

**Table 2:** List of calculated parameters of anchor rods.

Anchor type	Cross section area (${\mathrm{m}}^{2}$)	Modulus of elasticity, E (GPa)	Tensile strength (GPa)	Cement slurry bond stiffness (N/m/m)
Grouting	4.9*10^2^	200	0.445	2.0*10^–7^

**Table 3:** List of parameters of initial concrete spraying support.

$\gamma$ (kN/m^3^)	E (GPa)	Poisson’s ratio,$\mu$	Thickness (cm)
23	28	0.2	20

Usually, the tunnel support system consists of overrun support, initial support and secondary lining. The secondary lining mainly acts as a safety reserve, while the overrun support and initial support mainly bear most or even all of the rock pressure when the surrounding rock is deformed during construction. This paper simulates the laws of vault settlement sidewall convergence, the changes in axial force and bending moment, and the change in the safety factor of the initial support under different working conditions of the construction of a V-grade surrounding rock tunnel with an oversupport and initial support.

## Excavation simulation

In the construction of the broken rock section grade V, we need to follow the principles of weak blasting, short feed, less disturbance, early spraying and anchoring, diligent measurement, tight closure and early ring formation. To reduce the disturbance, it is necessary to optimize the construction parameters; the optimization of the parameters of the top heading and benching method (HB) and the CD method, which are commonly used in the construction of class V rock, lies in methods for determining the height of the steps. The height of the upper step is particularly important to determine; if the value is overly small, it directly affects the access of construction machinery and equipment, thus affecting the construction cycle. If the height of the upper platform is overly large, it affects the overall tunnel stability, surrounding rock stress release, tunnel support force, Tongzi tunnel construction, surrounding rock parameters, and support strength. We optimize the step height parameters in the construction of the top heading and benching method (HB) and the CD method. The height of the steps of the top heading and benching method (HB) is shown in Table 4, and the height of the steps of the CD method is shown in Table 5.

**Table 4:** Working conditions of the top heading and benching method (HB) construction.

Workmanship	Working conditions	Upper step height	Middle step height	Lower step height	Excavation footage per step
The top heading and benching method (HB)	M1	0.4H	0.4H	0.2H	5 m
	M2	0.45H	0.35H	0.2H	
	M3	0.5H	0.4H	0.1H	
	M4	0.55H	0.35H	0.1H	
	M5	0.6H	0.3H	0.1H

**Table 5:** Working conditions of the CD method construction.

Workmanship	Working conditions	Left (right) up step height	Left (right) lower step height	Excavation footage per step
CD method	M6	0.6H	0.4H	5 m
	M7	0.5H	0.5H	
	M8	0.4H	0.6H	

**Figure 6:**
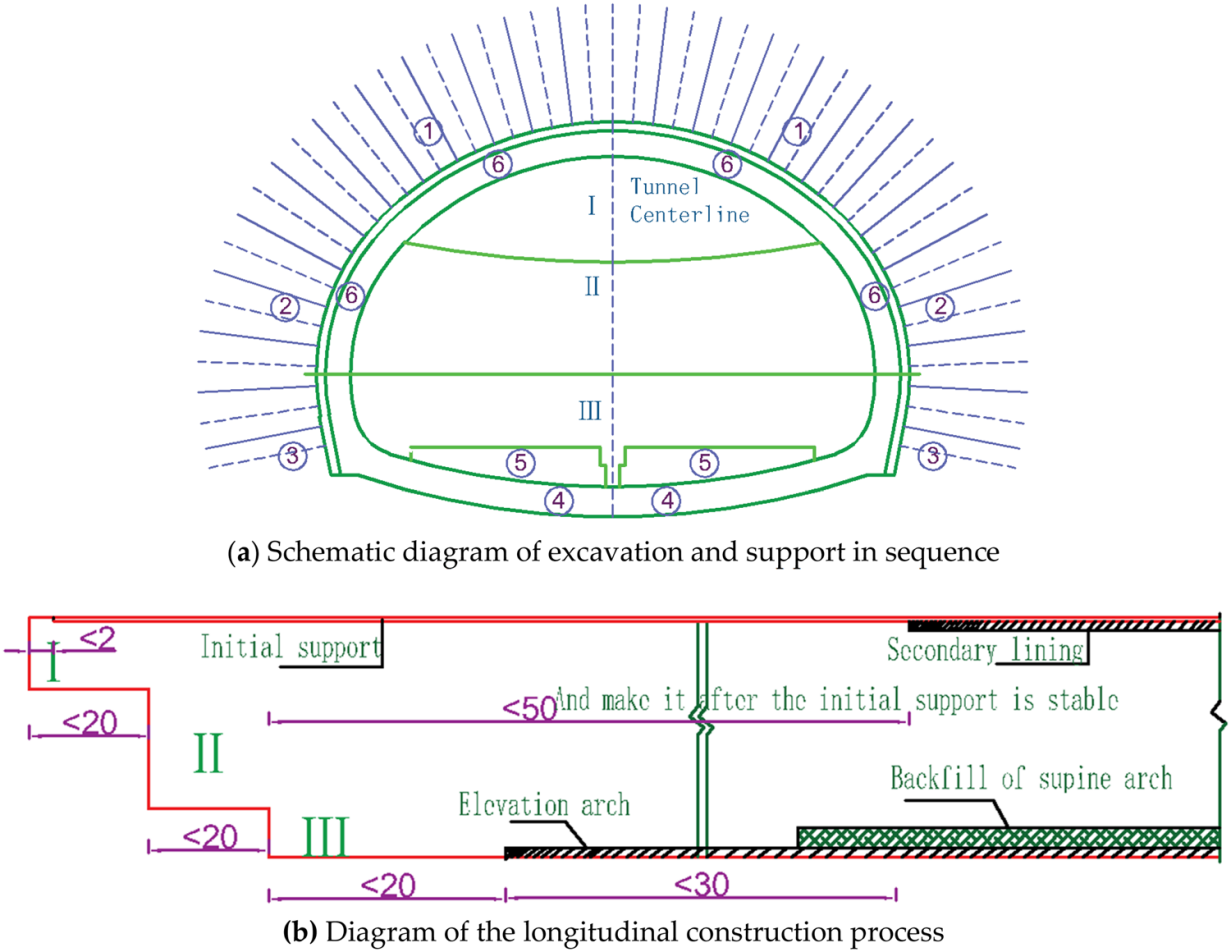
Schematic diagram of excavation and support by the top heading and benching method (HB).

The top heading and benching method (HB) construction site construction process is shown in Fig. 6a and b, and the steps are as follows. The upper Section I is excavated, initial support ① is applied → the trench is jumped, middle section II is excavated, and the corresponding initial support is applied ② → lower section III is excavated, and the corresponding initial support ③ is applied → after the initial support stabilizes, elevation arch ④ is applied → elevation arch backfill ⑤ is applied → overall moulded secondary lining ⑥ is applied.

## Calculation results and analysis

### Analysis of surrounding rock displacement results

**Figure 7:**
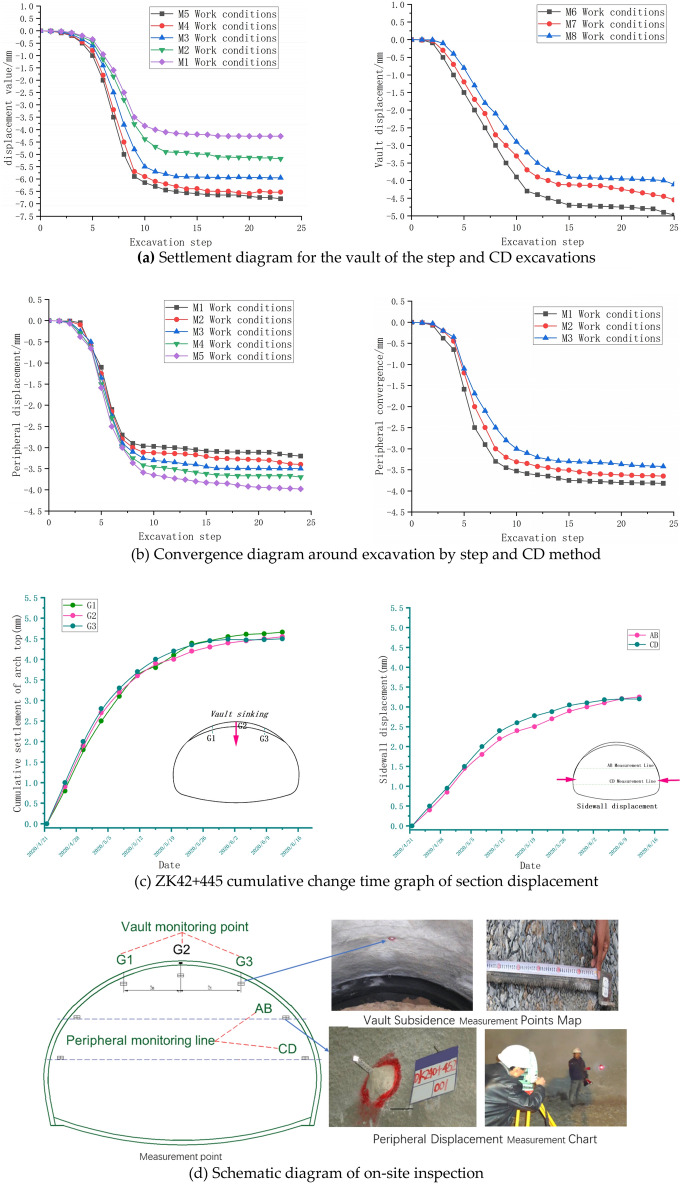
Settlement and peripheral displacement of the arch of excavation under different working conditions.

The surrounding rock displacement is of particular interest during the construction of the Neo-Australian method, as it is the most direct response to the overall forces in the surrounding rock^28^. The section at 20 m of the simulated tunnel is selected for monitoring, and the node at which the vault and level of the target section produce the maximum movement is analysed. The relationships between vault settlement and construction steps for the top heading and benching method (HB) and CD method under different working conditions are plotted in Fig. 7a, and the relationship between the maximum horizontal convergence and construction steps is shown in Fig. 7b. Site construction monitoring and measurement of displacement are shown in Fig. 7c. The difficulty of monitoring and measuring the subsidence of the arch crown is high. Due to the high height of the tunnel, it is also very difficult to hang a ruler on the arch crown, and the measurement accuracy is also poor. Therefore, all measurements are made without a ruler. When measuring the reading, it should be ensured that the initial reading is taken within 24 h after blasting. Set up a measuring section every 8 m. This measurement adopts the principle of free station setting with a total station to remotely measure the three-dimensional coordinates of points at different time periods. After processing, the three-dimensional displacement vector or relative convergence value of the measurement points are output, replacing traditional contact measurement for measuring the settlement of the arch crown and surrounding displacement. The layout of the measurement points is shown in Fig. 7d.

1. An analysis of Fig. 7a shows that there is a certain pattern of vault settlement in the construction of the top heading and benching method (HB) and the CD method. For the same height of the upper step, the settlement of the vault in the CD method is smaller than that in the top heading and benching method (HB). When the height of the upper step is 0.4H, the settlement value of the arch top of the CD method is 4.11 mm, and the settlement value of the arch top of the top heading and benching method (HB) is 6.8 mm. When the height of the upper step is 0.5H, the settlement value of the arch top of the CD method is 4.55 mm, and the settlement value of the arch top of the top heading and benching method (HB) is 5.95 mm. In the construction of the top heading and benching method (HB) and the CD method, the settlement of the vault increases with the increase in the height of the upper step; the settlement rate of the vault of the step construction is the largest at the seventh step of excavation and then decreases with the increase in the settlement rate of the excavation step. When changing from the M2 to the M3 working condition construction, the corresponding step of the excavation vault settlement change is relatively large, and in the eighth step construction, the vault settlement rate of the last three working methods becomes large. Among the (HB) heights of the CD method construction, the step height of the M7 working condition is transformed into the M8 working condition construction, and the increase in arch settlement is large.
2. Analysis of Fig. 7b shows that the convergence around the excavation of the top heading and benching method (HB) and the CD method is similar to the settlement of the vault. As the height of the upper step increases, the peripheral convergence increases. When the height of the upper step of the top heading and benching method (HB) is 0.4H, the convergence around the tunnel is 3.2 mm. When the height of the upper step is 0.45H, the convergence around the tunnel is 3.4 mm, and there is an increase of 0.2 mm, accounting for 5.8% of the maximum horizontal convergence. When the height of the upper step is 0.5H, the convergence around the tunnel is 3.5 mm. At the upper step height of 0.55H, the tunnel perimeter convergence is 3.7 mm, which is an increase of 0.2 mm from the 0.55H step height. When the height of the upper step is 0.6H, the convergence around the tunnel is 3.98 mm, which is 0.28 mm greater than the previous step, accounting for 7.0% of the maximum horizontal convergence. When the height of the upper left (right) step is 0.6H, the peripheral convergence is 3.82 mm. When the height of the left (right) upper step is 0.5H, the peripheral convergence is 3.65 mm. When the height of the left (right) upper step is 0.4H, the peripheral convergence is 3.42 mm, and the convergence variation is large. A comparison between the top heading and benching method (HB) and the CD method construction shows that the CD method construction has less influence on the stability of the surrounding rock under the same upper step height.
3. As shown in Fig. 7c, the selected target section vault settlement is accumulated to 4.55 mm, the maximum convergence around the tunnel is 3.27 mm, and the simulation results are consistent with the on-site monitoring data, reflecting the rationality of the simulation.

#### Analysis of the main stress results in the surrounding rock

The selection of the construction method in tunnel construction is particularly important, especially for the construction of Class V rocks, and the method directly affects the stability of the tunnel. The selection of the upper step height in the construction of the top heading and benching method (HB) and the CD method affects the stress release in the surrounding rock during construction. When the height of the upper step is determined, it is safe to construct the lower step. The simulated values are shown in Table 6 below for the stress analysis of the maximum and minimum values of the surrounding rock stress variations under different working conditions.

**Figure 8:**
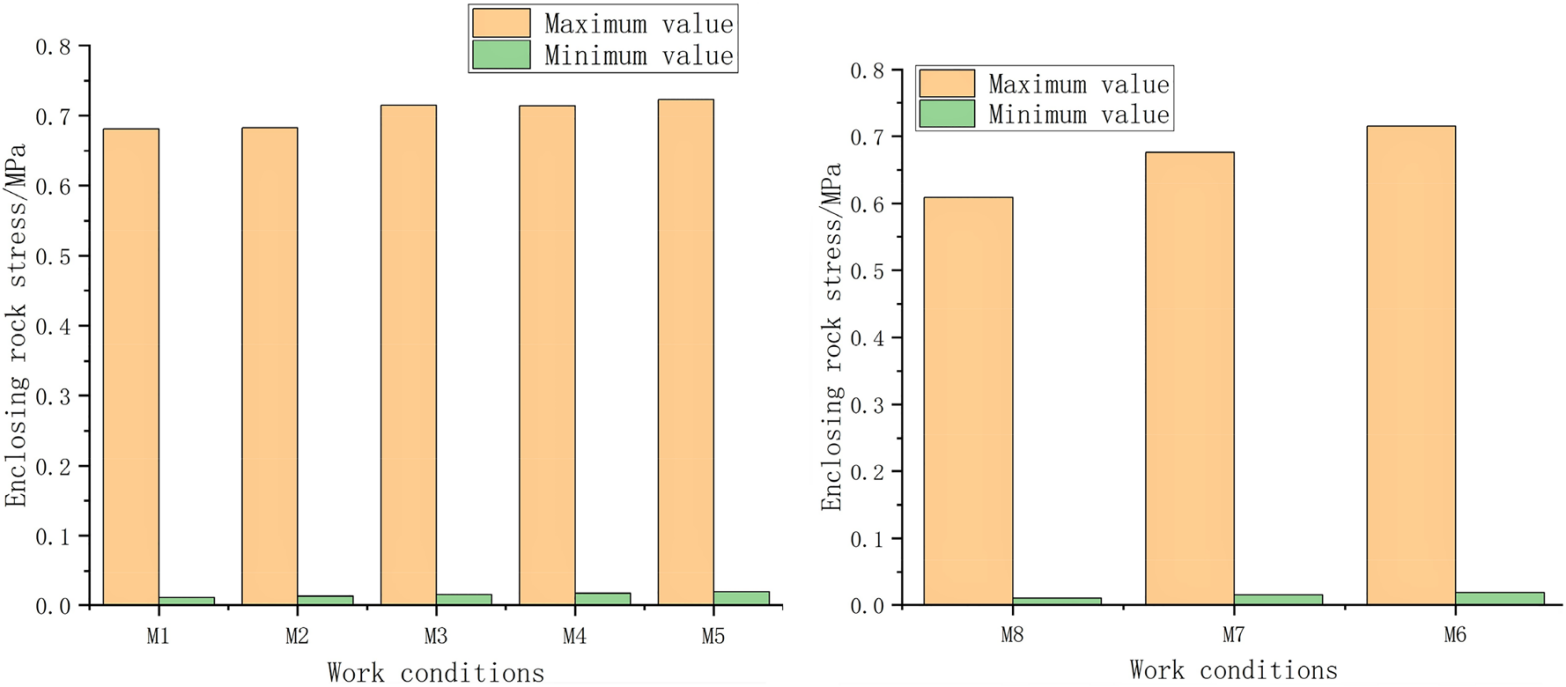
Comparison of the maximum and minimum values of the surrounding rock stress.

1. Data analysis of Table 6 is performed. The top heading and benching method (HB) of construction with the height of the upper step increases, and the maximum value of the surrounding rock stress increases. From M1 to M5, the maximum stress in the surrounding rock increases from 1.551 to 1.792 MPa, and the minimum stress in the surrounding rock increases. The maximum value of the surrounding rock stress changes the most in the construction of the (HB) from M2 to M3, and the maximum surrounding rock stress value increases from 1.583 to 1.763 MPa, with a difference of 0.18 MPa, accounting for 10.2% of the maximum surrounding rock stress, reflecting the great impact of the construction on the surrounding rock stress when the height of the upper step changes from 0.45 to 0.5H.
2. According to Fig. 8, as the height of the upper step increases, the surrounding rock stress increases. The maximum stress in the surrounding rock increases from 1.495 to 1.811 MPa when the construction of the CD method changes from M6 to M5; the difference is 0.316 MPa, accounting for 17.4% of the maximum surrounding rock stress, indicating that the construction has a great impact on the surrounding rock stress when the height of the upper step increases by 0.6 H from 0.5 H.

**Table 6:** Surrounding rock stresses with different working methods.

Workmanship	Working conditions	Equivalent rock stress (MPa)	
		Maximum value	Minimum value
The top heading and benching method (HB)	M1	1.551	0.011
	M2	1.583	0.013
	M3	1.763	0.015
	M4	1.792	0.017
	M5	1.811	0.019
CD method	M6	1.495	0.018
	M7	1.366	0.015
	M8	1.352	0.010

#### Analysis of the main stress results in the surrounding rock

**Figure 9:**
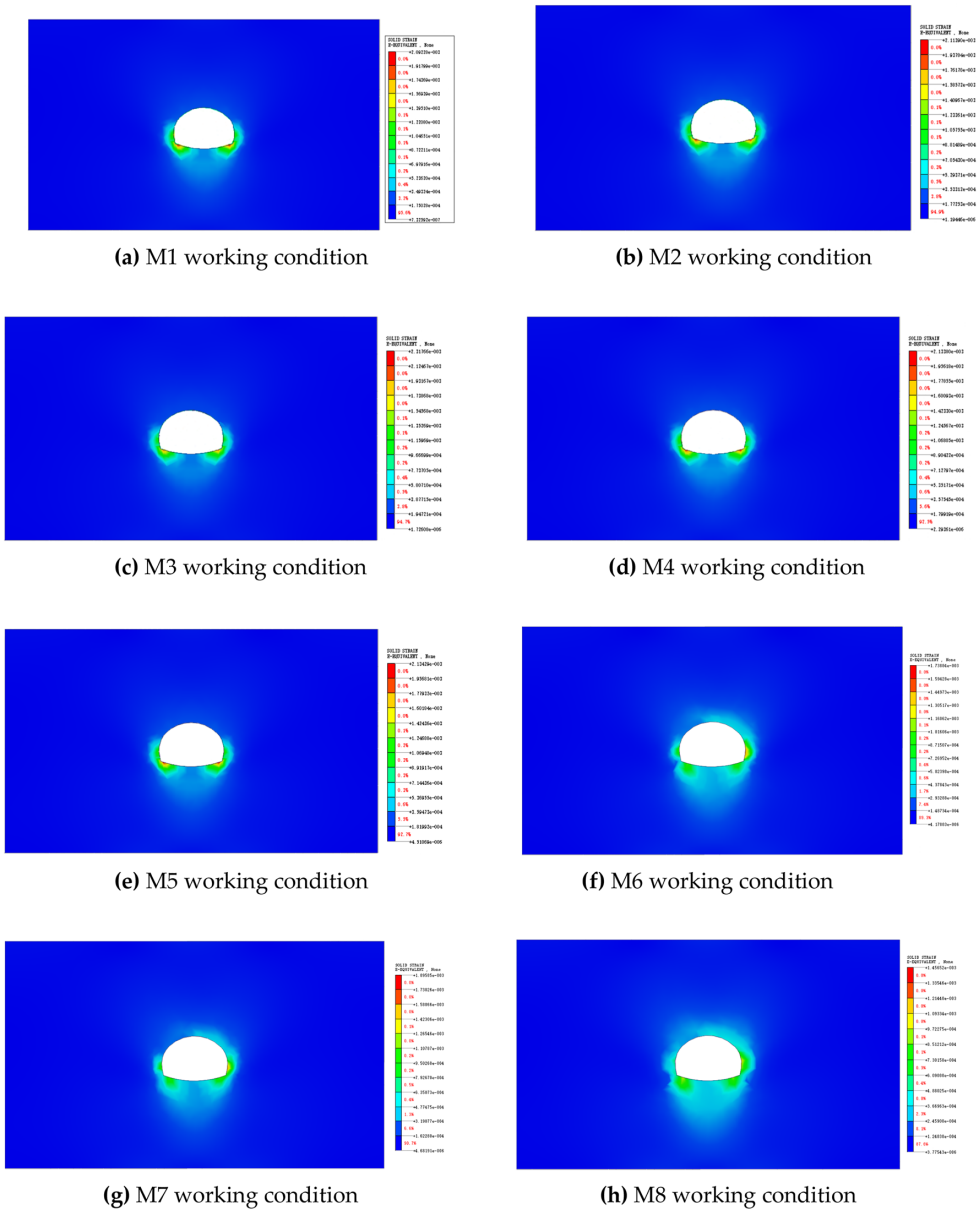
Comparison of the plastic zone of the excavated surrounding rock.

The tunnel distribution excavation necessarily causes multiple stress redistributions in the tunnel^29^. In tunnel construction, the mastery of the plastic zone of the surrounding rock is very important because the release of stress in the surrounding rock can be mastered by understanding the plastic zone of the surrounding rock. Figure 9 shows the plastic zone of the surrounding rock at 30 m after the excavation step to the target cross section under different working conditions.

According to Fig. 9a → b → c → d → e → f → g → h, with the increase in the height of the upper step, the plastic zone of the surrounding rock at 30 m after the target section increases with the increase in the height of the upper step. This finding suggests that the greater the height of the upper step is, the greater the disturbance of the rock surrounding the tunnel during construction, increasing its plastic zone. According to Fig. 9 above, under the same upper step height, the plastic zone in the same position of the surrounding rock is smaller in the construction of the CD method, suggesting that the construction of the CD method disturbs the surrounding rock less than the construction of the top heading and benching method (HB).

### Analysis of lining forces and structural safety factors

In tunnel construction, the force of the structure is very important, and it directly affects the safety of the construction. Therefore, the calculations of the axial force, bending moment and safety factor of the initial support are essential.

The axial force and bending moment of the structure and the safety factor of the structure can be calculated according to Eqs. (1)–(4)^30^.

The axial force and bending moment of the initial support are shown in Formulas (1) and (2):

1 $$ {\text{N}} = \frac{1}{2}{\text{E}}\left( {\upvarepsilon _{{{{\rm Inside}}}} +\upvarepsilon _{{{{\rm Outside}}}} } \right){\text{bh}} $$

2 $$ {\text{M}} = \frac{1}{12}{\text{E}}\left( {\upvarepsilon _{{{{\rm Outside}}}} -\upvarepsilon _{{{{\rm Outside}}}} } \right){\text{bh}}^{2} $$

The safety factor of the initial support is shown in Eqs. (3) and (4):

3 $$ {\text{KN}} \le \upvarphi \upalpha {\text{R}}_{{{\rm a}}} {\text{bh}} $$

4 $$ {\text{KN}} \le \upvarphi \frac{{1.75{\text{R}}_{{{\rm l}}} {\text{bh}}}}{{6{{{\text{e}}_{0} } \mathord{\left/ {\vphantom {{{\text{e}}_{0} } {{\text{h}} - 1}}} \right. \kern-0pt} {{\text{h}} - 1}}}} $$

where b is the section width, taken as 1 m; M is the bending moment; N is the axial force; E is the modulus of elasticity; h is the section thickness; α is the eccentric influence coefficient of axial force; φ is the longitudinal bending coefficient of the member; R_l_ is the tensile ultimate strength of concrete; R_a_ is the compressive ultimate strength of concrete; and K is the safety factor.

#### Axial forces

**Figure 10:**
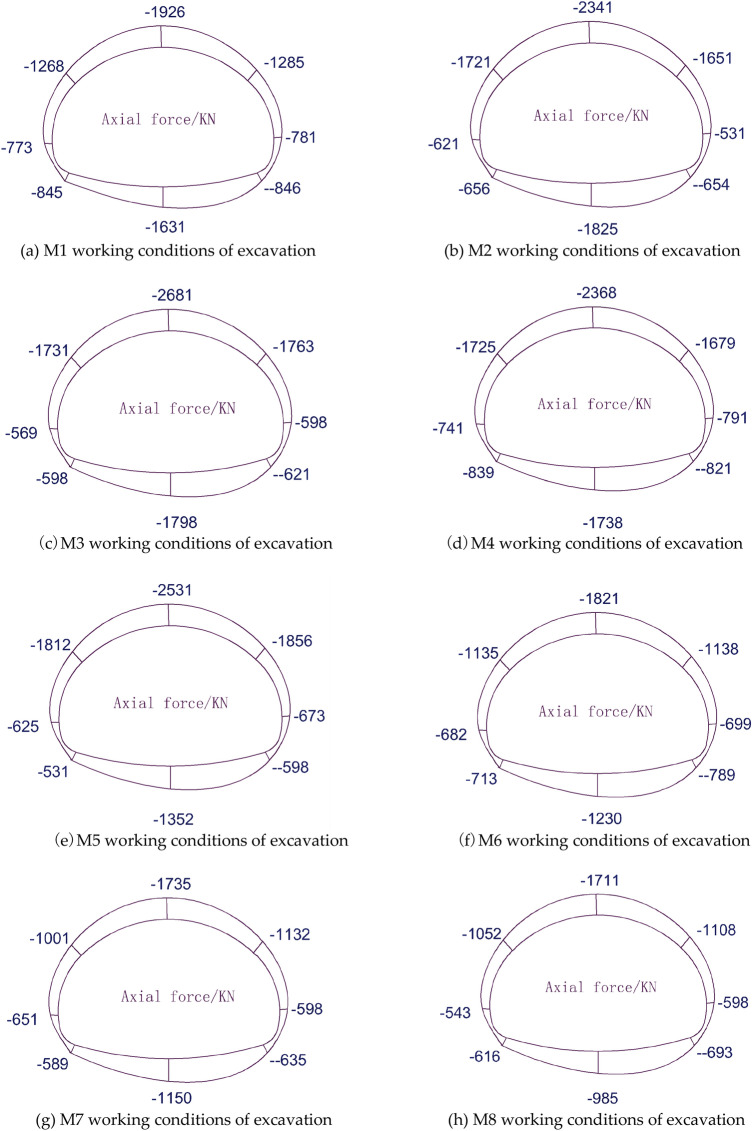
Axial force diagram for each operating condition.

The axial force of the initial support under each working condition is shown in Fig. 10 below.

As shown in Fig. 10a → b → c → d → e → f → g → h, the initial support forces are pressure, the forces on the left and right sides of the tunnel are basically symmetric, and the axial force at the top of the arch is the largest. With the increase in the height of the steps, the axial force at the same position generally appears to increase, the maximum axial force at the top of the arch of the top heading and benching method (HB) reaches − 2681 kN, the maximum axial force at the top of the arch of the CD method can reach − 1821 kN, with the top of the arch as the dividing point to both sides gradually decreasing, and the difference between the axial force on the left and right sides is not large.

#### Bending moment

**Figure 11:**
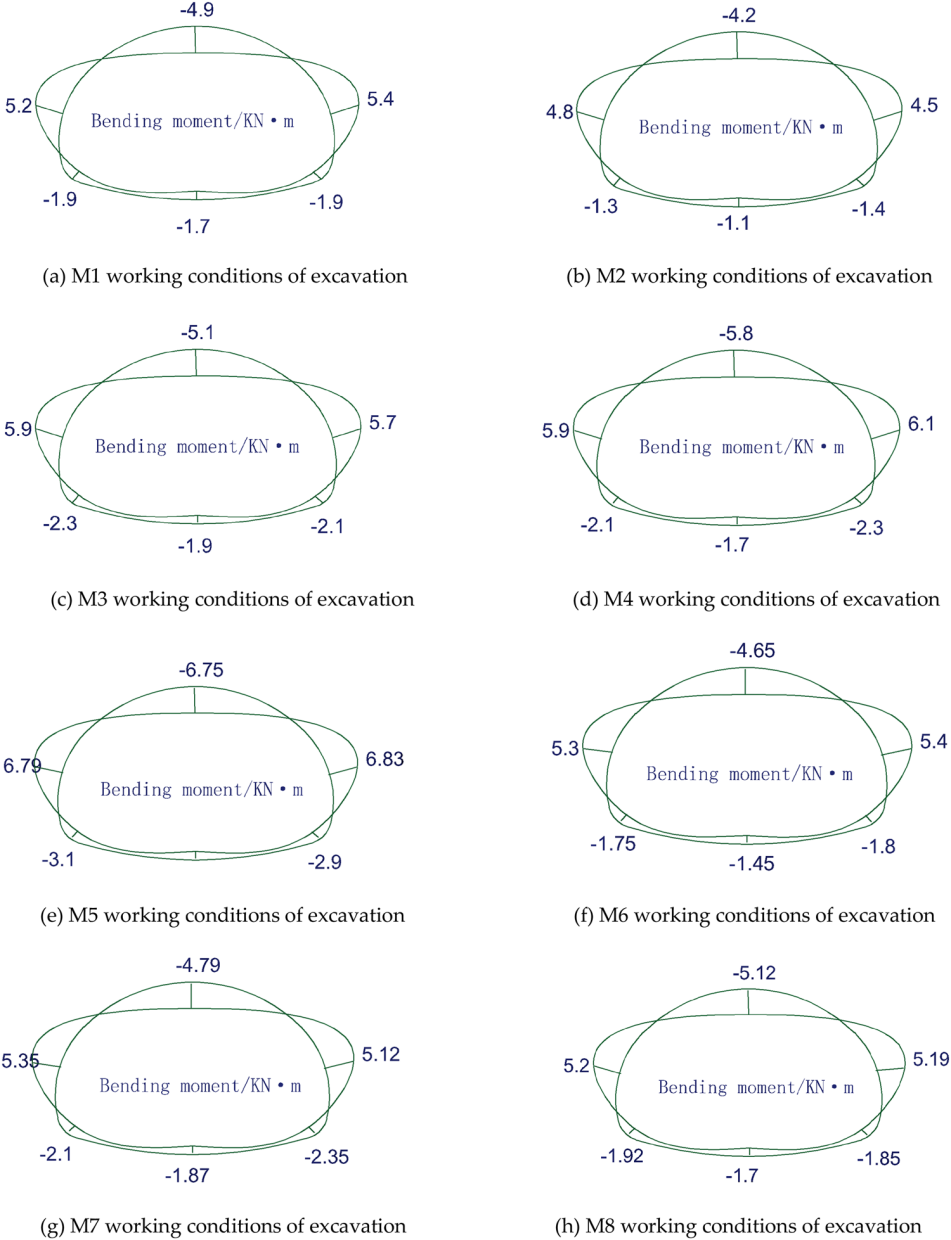
Bending moment diagram for each working condition.

The construction under different working conditions and the bending moment of the initial support under each working condition are shown in Fig. 11 below.

From the above Fig. 11a → b → c → d → e → f → g → h, the top heading and benching method (HB) and CD method of construction of different upper step height construction bending moments exist in a certain pattern. The maximum positive bending moment at the sidewall reaches 6.79 kN m. In the step construction method, the M2 working condition is designed with the minimum bending moment at the top of the arch and the shoulder of the arch at a height of 0.45H for the upper step, 0.35H for the middle step and 0.2H for the lower step. The maximum positive bending moment occurs at the sidewall for several conditions from the M1 to the M5 working condition. Therefore, the bending strength of the initial support of the sidewall area should be strengthened to avoid excessive bending moment, resulting in bending damage. During the construction of the CD method, the M6 and M7 working conditions are similar at the top of the arch; as the height of the upper step increases, the bending moment at the top and shoulder of the arch increases, and the maximum at the shoulder of the arch reaches 5.4 kN m. With the same upper step height, the bending moment at each point of the CD method is smaller than the bending moment of the top heading and benching method (HB).

#### Safety factor

The safety factor of each testing point was calculated, and the lowest safety factor was obtained at the vault. The safety factors of the construction vault under different working conditions are compiled in Table 7.

**Table 7:** Minimum safety factor under different working conditions of excavation.

Workmanship	Working conditions	Minimum safety factor
HB method	M1	5.80
	M2	4.78
	M3	4.17
	M4	4.72
	M5	4.42
CD method	M6	6.14
	M7	6.44
	M8	6.53

From the above table, it can be seen that as the height of the upper step increases, there is a certain pattern of safety factors at the top of the arch. The safety factor at the top of the arch decreases as the height of the upper step increases with the construction of the top heading and benching method (HB) and the CD method. The safety factor is reduced from 5.80 to 4.42 for the HB method M1 to M5, and the safety factor is reduced from 4.78 to 4.17 under the M2 to M3 working conditions, which is 0.61. The CD method has a higher safety factor than the top heading and benching method (HB) for the same upper step height construction. The safety factors of the M1 and M2 working conditions in top heading and benching method (HB) construction are not much different and are the largest among the first five working conditions; the safety factors of the M7 and M8 working conditions in CD method construction are similar.

## Conclusion

By simulating the construction of the top heading and benching method (HB) and CD method with different step heights through MIDAS/GTS NX, the tunnel vault settlement, peripheral convergence, surrounding rock stress, surrounding rock plastic zone, bending moment, axial force applied to the initial support and the safety factor were analysed, and the following conclusions were obtained.

1. Aspects of the surrounding rock displacement: Compared with the construction of the CD method at the same upper step height, the arch settlement and peripheral convergence of the CD method construction were smaller; with the increase in upper step height, the arch settlement and peripheral convergence increased; therefore, to reduce the surrounding rock disturbance, the upper step height construction should be reduced as much as possible.
2. Initial support aspects: The tunnel should be supported in time after excavation because the timely application of support could close the surrounding rock, thus reducing the surrounding rock disturbance; this phenomenon was conducive to the stability of the surrounding rock. The maximum positive bending moment occurred at the sidewall under several working conditions; thus, the bending strength of the initial support at the sidewall should be strengthened to avoid excessive bending moment, resulting in bending damage.
3. By analysing the tunnel vault settlement, peripheral convergence, surrounding rock stress, surrounding rock plastic zone, bending moment and axial force characteristics applied to the initial support, the safety factor was determined from the numerical simulation results. The optimal step height for the top heading and benching method (HB) excavation was 0.45H for the upper step, 0.35H for the middle step and 0.2H for the lower step. The best step height in the CD method excavation was left (right) upper step height of 0.5H and left (right) lower step height of 0.5H.

## Data Availability

All data generated or analysed during this study are included in this article.
